# Mapping evidence on women’s knowledge and practice of breast self-examination in sub-Saharan Africa: a scoping review protocol

**DOI:** 10.1186/s13643-019-1254-7

**Published:** 2020-01-06

**Authors:** Roseline H. Udoh, Monica Ansu-Mensah, Mohammed Tahiru, Vitalis Bawontuo, Desmond Kuupiel

**Affiliations:** 1 Faculty of Health and Allied Sciences, Catholic University College of Ghana, Fiapre, Sunyani, Ghana; 2 Department of Public Health Medicine, School of Nursing and Public Health, University of KwaZulu-Natal, Durban, South Africa; 3 Research for Sustainable Development Consult, Sunyani, Ghana

**Keywords:** Women, Breast cancer, Breast self-examination, Knowledge, Practice, Early detection, and Sub-Saharan Africa

## Abstract

**Background:**

Globally, breast cancer is the most common malignant condition in women. Breast self-examination practice following correct procedure potentially can lead to early detection of breast abnormalities. We propose to systematically chart literature and examine the scope of evidence on women’s knowledge and practice of breast self-examination in sub-Saharan Africa (SSA).

**Methods:**

Our scoping review methods will be guided by the framework proposed by Arksey and O'Malley, Levac et al. and Joanna Briggs Institute guidelines. Literature searches will be conducted in the following electronic databases (from 2008 onwards): PubMed/MEDLINE, Scopus, Web of Science, CINAHL, PsycINFO and Health Sources. Grey literature will be identified through searching dissertation databases, Google Scholar and governmental databases. Two reviewers will screen all citations and full-text articles We will abstract data, organise them into themes and sub-themes, summarise them and report the results using a narrative synthesis. The study methodological quality (or bias) will be appraised using a mixed-method appraisal tool.

**Discussion:**

The findings from the scoping review will contribute to obtain an understanding of the women’s knowledge and practice of breast self-examination in sub-Saharan Africa, and will likely reveal the depth of evidence helping to identify gaps for future research. Results will be published in a peer-reviewed journal. Implications for clinical practice and health policy will be discussed.

## Background

Breast cancer (BC) is the most common malignant condition in women and the second most common malignant condition worldwide [1], and the leading cause of cancer-related deaths among women [2, 3]. In 2012, BC alone accounted for 1.7 million (12.1%) of the total 14.1 million women diagnosed with cancer worldwide [3–5]. Of the 12.1% of women diagnosed with BC, globally in 2012, about 56.8% were diagnosed in low- and middle-income countries (LMICs) [4]. Over 522,000 BC deaths among women were recorded in the same year, and the majority of these deaths occurred in sub-Saharan Africa (SSA) [6, 7]. Global projections on BC burden indicates that over 19 million new cases will occur by the year 2020 and 2025 [6, 8–11]. Of these 19.7 million BC new cases, 10.6 million cases will occur in LMICs, accounting for over 1 million BC new cases per year [5, 8–10]. At the same time, global BC mortality projections also show that 8.5 million women will die, of which 3.9 million of these deaths will occur in LMICs causing more than 1.5 million premature and preventable deaths [4, 8].

These projections on BC morbidity and mortality are worrying and require global actions to prevent BC and improve the health outcomes of BC patients. To enable early detection of BC and prompt linkage to care, screening examination of the breast such as breast self-examination (BSE) and/or clinical breast examination (CBE) are highly essential. Regular screening of the breast facilitates recognition of breast abnormalities such as the lesion or lump as early as possible and link patients to various treatment options and supportive management [2, 12, 13]. It has been reported that there are more treatment choices for BC patients and a better chance for survival associated with early BC detected [2]. A survival rate of approximately 93% or more has been reported for women whose BC is detected at an early stage (first 5 years) [2]. For early detection of breast lesion or lump, an examination of the breast should be done either by the individual BSE or by health personnel CBE [14, 15]. However, due to several reasons such as poor health-seeking behaviour, poor access to healthcare, late reporting of BC to health facilities and limited availability of cancer diagnostic and treatment facilities, particularly in SSA, the concept of BSE and reporting of breast abnormalities for CBE is vital. Knowledge and practice of BSE following the correct procedure has been demonstrated to be essential for self-detection of any breast abnormalities and reporting to health facilities for prompt initiation of clinical interventions such as CBE, diagnosis and treatment [12, 14, 15]. This proposed scoping review will, therefore, aim to chart literature and examine the scope of evidence on the knowledge and practice of BSE among women in SSA.

## Methods

### Overview

Scoping review maps a range of literature existing around a research field of interest to identify gaps for future research [16, 17]. A scoping review is also helpful in determining the need and value of a primary study or systematic review [16]. This study will be guided by the 2005 Arksey and O’Malley scoping review framework incorporating the Levac et al. 2010 recommendation [16, 18] which stipulates the following: identification of the research questions; identification of relevant studies; study selection; charting and collating of data, summarising and reporting findings, and the 2015 Joanna Briggs Institute guidelines [19]. The present protocol is being reported in accordance with the reporting guidance provided in the Preferred Reporting Items for Systematic Reviews and Meta-Analyses Protocol (PRISMA-P) statement [20] (see checklist in Additional file 1).

### Identifying the research question

Our overall research question will be: What are the evidence on women’s knowledge and practice of BSE in SSA?

The sub-review questions will be as follows:

- What evidence exists on the knowledge of BSE among women in SSA?
- What evidence exists on the practice of BSE among women in SSA?
- Is there evidence linking early BC detection among women to BSE in SSA?

### Information sources and search strategy

A comprehensive search for relevant primary studies published in peer-reviewed journals on women’s knowledge and practice of BSE will be done in the following databases (from 2008 onwards): PubMed, SCOPUS, Web of Science, Google Scholar and EBSCOhost (Academic Search Complete, MEDLINE, CINAHL, PsycINFO and Health Sources) using a combination of appropriate keywords and index terms. The keywords for the online database search will include “women” OR “woman” OR “female” OR “mothers” AND “breast” OR “Breast” OR “breast cancer” OR “cancer” OR“carcinoma” OR “lumps” OR “tumour”OR “neoplasms” OR “tumor” OR “malignancy” OR “benign” AND “breast self-examination” OR “self-breast examination”, OR “examination” OR “screening” AND “knowledge” OR “practice” OR“early detection” OR “diagnosis”, “sub-saharan africa” OR “Africa”. We will also search by country names to enable us to access all relevant articles. Medical Subject Heading (MeSH) terms in all fields will also be included to enable the identification of all relevant studies. Limitations on study design, date of publication and language will be removed during the search for potential articles in the databases. Relevant grey literature like unpublished studies, thesis and dissertations will also be included. We will additionally search for relevant literature from the World Health Organization and governmental websites, and from the reference list of included articles. A detailed searched record will be documented as follows: date of search, search engine, keywords and number of retrieved publications and the number of eligible studies. A draft search strategy for PubMed/MEDLINE is provided in Additional file 2.

### Eligibility criteria

To be included in the scoping review, an article will have to measure or focus on knowledge and practice of BSE in the conceptual framework. Peer-reviewed journal articles and unpublished reports will be included if they are published between the period of 2008–to date of final search, are written in English, involved adult women participants (18 years and above), described evidence in the context of early detection of BC with BSE and are conducted in SSA countries. Quantitative, qualitative and mixed-method studies will be included in order to consider different aspects of measuring knowledge and practices. Papers will be excluded if they did not fit into the conceptual framework of the study, focused on men, CBE, BC detection using mammography or treatment of BC. In addition, reviews will be excluded.

### Selection of sources of evidence

A comprehensive title screening will be done by the principal investigator and all eligibility studies imported to endnotes X7 library created for the study. The library will then be cleaned by thoroughly examining and deleting duplicate articles. Following this process, the final endnote library will be shared with co-reviewers for the next stage of the screening process. Two independent reviewers will perform both abstract and full-text screening using the eligibility criteria. Disagreements between reviewers’ responses at the abstract screening stage will be addressed via deliberations with the co-reviewer to reach a consensus. Also, disagreements at the full-text screening stage between reviewers for this proposed scoping review will be addressed by consulting a third reviewer. We will also calculate the inter-rater agreement (Cohen’s kappa co-efficient (*k*) statistics) between reviewers’ responses as well as McNemar’s chi-square statistic. In the case where a full-text article cannot be retrieved or is not accessible from the online databases, we will seek assistance from the Catholic University College library, University of Ghana library or the University of KwaZulu-Natal library. We will also write to the authors to request full-text articles not accessible online. A flow chart showing details of studies included and excluded at each stage of the study selection process will be provided [21].

### Charting the data

We will extract relevant information from the included studies to enable us to answer the proposed scoping review question. To ensure accuracy and consistency in the data that will be extracted, two reviewers will independently pilot the data extraction form using 10% of the included studies in parallel. The data extraction form will be adjusted before its final use based on feedback from the reviewers. We will also keep the data extraction form updated until all relevant information has been extracted from the included studies. Table 1 illustrates the data extraction form that will be used for the proposed scoping review.

**Table 1:** Data extraction form

Author and date of publication	
Study title	
Study aim/objective	
Type of study design	
Study setting (country)	
Geography setting (rural/urban)	
Study population	
Number of study participants	
Study findings	
Significant findings	
Conclusions	

### Collating, summarising, and reporting the results

We will present information about the included articles that align to the objective and questions of the review. We will extract all relevant data from the eligible studies manually using the piloted data extraction form designed in a tabular format to answer the review question. We will also organise all the data extracted into themes and sub-themes, summarise them and report the results using a narrative approach, tables and appropriate figures. Other emerging themes will also be reported. The PRISMA extension for scoping review checklist will be used to guide the result paper of this proposed study. Subsequently, a systematic review and meta-analysis may be conducted using quantitative data if warranted.

### Quality appraisal

The methodological quality of all included studies will be assessed using the Mixed Method Quality Appraisal Tool (MMAT) version 2018. The included studies will be appraised by reading thoroughly each included article and responding appropriately to the questions for the type of study design as prescribed by the MMAT. To avoid bias, two reviewers will independently perform the quality assessment, and in case of any disagreement, a third reviewer will be contacted to resolve the discrepancies. The score of each appraised article will be calculated and the results interpreted as ≤ 50% low quality, 51–75% average quality and 76–100% high quality [22].

## Discussion

This scoping review is to map literature and examine the scope of evidence on the knowledge and practice of BSE among women in SSA. There has been a rise in morbidity and mortality due to BC in recent times [12, 23, 24]. Late reporting or detection of BC is seen as contributing to high mortality in SSA [12]. Practicing BSE by women can potentially help early BC detection at home prior to CBE, diagnosis and prompt initiation of treatment. Therefore, literature published from 2008 to the last search date in 2019 will be searched and screened and the included articles synthesised to enable retrieval of current information on women’s knowledge and practice of BSE in SSA. This will also enable us to know the extent of recent (from 2008 to 2019) research activity on BSE among women in SSA and identify gaps for recommendations on future research. Due to the lack of expertise for other languages such as French, Spanish, German, Arabic and others, we will limit the publication language to only English during the abstract and full-text screening stages. Notwithstanding this, we anticipate retrieving many articles for this review. Although the inclusion of methodology appraisal of included articles is not mandatory for a scoping review study, we will include it to help report on the risk of bias and to inform the quality of the evidence.

The implications, strengths and limitations will be adequately reported. We anticipate that the findings from this proposed scoping review will help improve BSE among women by key stakeholders, reveal research gaps to inform future research to provide evidence-based solutions to improve BSE among women, and contribute to early BC detection, diagnosis and treatment of BC in SSA. The finding of this proposed study may also inspire countries in SSA to advocate for less-expensive and simple diagnostic tests for use in resource-limited settings or self-testing to facilitate early detection of BC at primary healthcare clinics and by individuals at homes to reduce the impact BC in SSA.

## Supplementary information

**Additional file 1.** PRISMA-P 2015 Checklist.

**Additional file 2.** Pilot search in PubMed electronic database.

## Data Availability

We have duly cited all studies, and data is presented in the form of references.

## Abbreviations

BC: Breast cancer
BSE: Breast self-examination
LMIC: Low- and middle-income countries
SSA: Sub-Saharan Africa

## References

[1] Rivera-Franco MM, Leon-Rodriguez E (2018). Delays in breast cancer detection and treatment in developing countries. Breast Cancer.

[2] Milgard C. Breast Center USA2019 [cited 2019 8/4/2019]. Available from: http://www.carolmilgardbreastcenter.org/early-detection.

[3] Angaher. An overview of breast cancer epidemiology, risk factors, pathophysiology and cancer risk reduction. 2017;1:00019.

[4] Are C, Rajaram S, Are M, Raj H, Anderson BO, Chaluvarya Swamy R, et al. A review of global cancer burden: trends, challenges, strategies, and a role for surgeons. 2013;107(2):221–6. 10.1002/jso.23248 22926725

[5] Anderson BO. Breast cancer—thinking globally: American Association for the Advancement of Science; 2014.

[6] Fletcher C, Bridge J, Hogendoorn P, Mertens FJWHO (2013). WHO classification of tumours of soft tissue and bone (IARC WHO classification of tumours).

[7] Sinn H-P, Kreipe HJBC. A brief overview of the WHO classification of breast tumors. 2013;8(2):149–54. 10.1159/000350774 PMC3683948 24415964

[8] Gradishar William J., Anderson Benjamin O., Blair Sarah L., Burstein Harold J., Cyr Amy, Elias Anthony D., Farrar William B., Forero Andres, Giordano Sharon Hermes, Goldstein Lori J., Hayes Daniel F., Hudis Clifford A., Isakoff Steven J., Ljung Britt-Marie E., Marcom P. Kelly, Mayer Ingrid A., McCormick Beryl, Miller Robert S., Pegram Mark, Pierce Lori J., Reed Elizabeth C., Salerno Kilian E., Schwartzberg Lee S., Smith Mary Lou, Soliman Hatem, Somlo George, Ward John H., Wolff Antonio C., Zellars Richard, Shead Dorothy A., Kumar Rashmi (2014). Breast Cancer Version 3.2014. Journal of the National Comprehensive Cancer Network.

[9] Distelhorst SR, Cleary JF, Ganz PA, Bese N, Camacho-Rodriguez R, Cardoso F (2015). Optimisation of the continuum of supportive and palliative care for patients with breast cancer in low-income and middle-income countries: executive summary of the Breast Health Global Initiative, 2014. Lancet Oncol.

[10] Ferlay J, Soerjomataram I, Ervik M, Dikshit R, Eser S, Mathers C, et al. GLOBOCAN 2012 v1. 0, Cancer incidence and mortality worldwide: IARC CancerBase No. 11. 2013. 2014. 10.1002/ijc.29210 25220842

[11] Cumber SN, Nchanji KN, Tsoka-Gwegweni JMJSAJoGO. Breast cancer among women in sub-Saharan Africa: prevalence and a situational analysis. 2017;9(2):35-37.

[12] Black E, Richmond R (2019). Improving early detection of breast cancer in sub-Saharan Africa: why mammography may not be the way forward. Global Health.

[13] Hassan LM, Mahmoud N, Miller AB, Iraj H, Mohsen M, Majid J (2015). Evaluation of effect of self-examination and physical examination on breast cancer. Breast.

[14] Yip CH, Smith RA, Anderson BO, Miller AB, Thomas DB, Ang ES, et al. Guideline implementation for breast healthcare in low-and middle-income countries: Early detection resource allocation. 2008;113(S8):2244–56. 10.1002/cncr.23842 18837017

[15] Echavarria MI, Anderson BO, Duggan C, Thompson B (2014). Global uptake of BHGI guidelines for breast cancer. Lancet Oncol.

[16] Arksey H, O'Malley L (2005). Scoping studies: towards a methodological framework. Int J Soc Res Methodol.

[17] Peters MD, Godfrey CM, Khalil H, McInerney P, Parker D, Soares CB (2015). Guidance for conducting systematic scoping reviews. Int J Evid Based Healthc.

[18] Levac D, Colquhoun H, O’Brien KK (2010). Scoping studies: advancing the methodology. Implement Sci.

[19] Institute JB. Joanna Briggs Institute reviewers’ manual: 2015 edition/supplement: Methodology for JBI Scoping Reviews Adelaide: The Joanna Briggs Institute; 2015.

[20] Moher D, Shamseer L, Clarke M, Ghersi D, Liberati A, Petticrew M, et al. Preferred reporting items for systematic review and meta-analysis protocols (PRISMA-P) 2015 statement. Syst Rev. 2015;4(1) 10.1186/2046-4053-4-1. 10.1186/2046-4053-4-1 PMC4320440 25554246

[21] Moher D, Liberati A, Tetzlaff J, Altman DG, The PRISMA Group. PRISMA 2009 Flow Diagram. 2009;6(2009):1000097.

[22] Kuupiel D, Bawontuo V, Mashamba-Thompson TP. Mapping evidence on tuberculosis active case finding policies, strategies, and interventions for tuberculosis key populations: a. systematic scoping review protocol. Systematic Reviews. 2019;8(1):162. 10.1186/s13643-019-1098-1 PMC6617702 31291993

[23] Kisiangani J, Baliddawa J, Marinda P, Mabeya H, Choge JK, Adino EO (2018). Determinants of breast cancer early detection for cues to expanded control and care: the lived experiences among women from Western Kenya. BMC Womens Health.

[24] Braun Virginia, Clarke Victoria, Hayfield Nikki, Terry Gareth (2019). Thematic Analysis. Handbook of Research Methods in Health Social Sciences.

