# Supplementary material for: Mapping evidence on women’s knowledge and practice of breast self-examination in sub-Saharan Africa: a scoping review protocol
Source: Syst Rev. 2020 Jan 6;9:2. doi: 10.1186/s13643-019-1254-7 (PMC6945542; doi:10.1186/s13643-019-1254-7)
Supplement: Supplementary file 2 — Additional file 2. Pilot search in PubMed electronic database. [file 13643_2019_1254_MOESM2_ESM.docx]

**Additional file 2:** Pilot search of PubMed electronic database

| **Date** | **Database** | **Keywords** | **Search results** |
| --- | --- | --- | --- |
| 27/17/2019 | PubMed | (((((((((((((((((("women"[MeSH Terms] OR "women"[All Fields]) OR ("women"[MeSH Terms] OR "women"[All Fields] OR "woman"[All Fields])) OR ("mothers"[MeSH Terms] OR "mothers"[All Fields])) OR ("female"[MeSH Terms] OR "female"[All Fields])) AND ("breast"[MeSH Terms] OR "breast"[All Fields])) OR ("breast neoplasms"[MeSH Terms] OR ("breast"[All Fields] AND "neoplasms"[All Fields]) OR "breast neoplasms"[All Fields] OR ("breast"[All Fields] AND "cancer"[All Fields]) OR "breast cancer"[All Fields])) OR ("neoplasms"[MeSH Terms] OR "neoplasms"[All Fields] OR "cancer"[All Fields])) OR ("carcinoma"[MeSH Terms] OR "carcinoma"[All Fields])) OR lump[All Fields]) OR ("tumour"[All Fields] OR "neoplasms"[MeSH Terms] OR "neoplasms"[All Fields] OR "tumor"[All Fields])) OR ("tumour"[All Fields] OR "neoplasms"[MeSH Terms] OR "neoplasms"[All Fields] OR "tumor"[All Fields])) OR ("neoplasms"[MeSH Terms] OR "neoplasms"[All Fields] OR "malignancy"[All Fields])) AND ("breast self-examination"[MeSH Terms] OR ("breast"[All Fields] AND "self-examination"[All Fields]) OR "breast self-examination"[All Fields] OR ("self"[All Fields] AND "breast"[All Fields] AND "examination"[All Fields]) OR "self breast examination"[All Fields])) OR ("breast self-examination"[MeSH Terms] OR ("breast"[All Fields] AND "self-examination"[All Fields]) OR "breast self-examination"[All Fields] OR ("breast"[All Fields] AND "self"[All Fields] AND "examination"[All Fields]) OR "breast self examination"[All Fields])) OR ("diagnosis"[Subheading] OR "diagnosis"[All Fields] OR "screening"[All Fields] OR "mass screening"[MeSH Terms] OR ("mass"[All Fields] AND "screening"[All Fields]) OR "mass screening"[All Fields] OR "screening"[All Fields] OR "early detection of cancer"[MeSH Terms] OR ("early"[All Fields] AND "detection"[All Fields] AND "cancer"[All Fields]) OR "early detection of cancer"[All Fields])) OR ("physical examination"[MeSH Terms] OR ("physical"[All Fields] AND "examination"[All Fields]) OR "physical examination"[All Fields] OR "examination"[All Fields])) AND ("knowledge"[MeSH Terms] OR "knowledge"[All Fields])) OR ("Practice (Birm)"[Journal] OR "practice"[All Fields])) AND ("africa"[MeSH Terms] OR "africa"[All Fields]) | 26840 |
